# Prediction Table and Nomogram as Tools for Diagnosis of Papillary Thyroid Carcinoma: Combined Analysis of Ultrasonography, Fine-Needle Aspiration Biopsy, and BRAF V600E Mutation

**DOI:** 10.1097/MD.0000000000000760

**Published:** 2015-05-29

**Authors:** Seo Ki Kim, Jun Ho Lee, Jung-Woo Woo, Inhye Park, Jun-Ho Choe, Jung-Han Kim, Jee Soo Kim

**Affiliations:** From the Division of Breast and Endocrine Surgery (SKK, J-WW, IP, J-HC, J-HK, JSK), Department of Surgery, Samsung Medical Center, Sungkyunkwan University School of Medicine, Seoul; and Division of Breast and Endocrine Surgery (JHL), Department of Surgery, Samsung Changwon Hospital, Sungkyunkwan University School of Medicine, Changwon, South Korea.

## Abstract

Although ultrasonography (US)-guided fine-needle aspiration biopsy (FNAB) is the most reliable diagnostic modality for evaluating thyroid nodules, 10% to 40% of FNAB samples yield indeterminate findings. The BRAF V600E mutation, a highly specific molecular marker for papillary thyroid carcinoma (PTC), well known for its prognostic value, has dubious diagnostic value because of its low sensitivity. Novel strategies are clearly needed to distinguish PTC, which represents the majority of thyroid malignancies, from other thyroid nodules.

The records of 3297 patients with surgically proven PTC were retrospectively reviewed. A prediction table and nomogram were designed using a combination of diagnostic parameters for US, FNAB, and the BRAF V600E mutation. For the nomogram, parameters were proportionally assigned 0 to 100 points according to their regression coefficient for PTC.

The probability of PTC for thyroid nodules with intermediate-risk (IR) US and atypia of undetermined significance/follicular lesion of undetermined significance (AUS/FLUS) FNAB was significantly dependent on BRAF V600E mutation status based on our prediction table (negative, 29.2% vs positive, 87.5%; *P* < 0.001). By our nomogram, the probability of PTC for thyroid nodules with IR US, AUS/FLUS FNAB, and positive BRAF V600E mutation was approximately 85% to 90%.

We strongly recommend preoperative evaluation of the BRAF V600E mutation in indeterminate thyroid nodules. The prediction table and nomogram developed in this study could help clinicians and patients easily assess the probability of PTC in the preoperative period.

## INTRODUCTION

Thyroid nodules are common in the general population. With the widespread use of high-resolution ultrasonography (US), the incidence of thyroid nodules in randomly selected individuals is estimated to be 19% to 67%.^1–4^ Among incidentally detected thyroid nodules, the vast majority are benign (BN) and can be managed conservatively. The remaining malignant (MN) cases found in approximately 5% to 15% of incidental detections need to be managed surgically.^5–8^ Papillary thyroid carcinoma (PTC) is the most common pathologic category of thyroid cancer.^9–11^ Although PTC follows an indolent and curable disease course, many patients experience disease persistence, recurrence, and even mortality^12–15^ The problem lies in defining which nodules to leave, which to remove, and what to do in case of an indeterminate thyroid nodule in order to avoid unnecessary examinations and surgery. Therefore, early and accurate diagnosis of PTC is the top priority for incidentally detected thyroid nodules.

Currently, US-guided fine-needle aspiration biopsy (FNAB) with cytological analysis is the most reliable diagnostic modality for evaluating incidentally detected thyroid nodules.^15–18^ The sensitivity and specificity of FNAB are reported as 65% to 98% and 72% to 100%, respectively.^18^ The Bethesda System for Reporting Thyroid Cytopathology (TBST) was developed to provide uniform terminology and diagnostic criteria.^19^ The TBST comprises 6 diagnostic categories with unique risks of malignancy and offers recommendations for clinical management. As reported in the previous studies, 10% to 40% of all FNAB samples yield indeterminate cytological findings.^20,21^ On the basis of the TBST, the risks of malignancy in indeterminate cytology findings are nonnegligible: 1% to 4% for nondiagnostic/unsatisfactory (ND/UNS), 5% to 15% for atypia of undetermined significance/follicular lesion of undetermined significance (AUS/FLUS), 15% to 30% for follicular neoplasm/suspicious follicular neoplasm (FN/SFN), and 60% to 75% for suspicious for malignancy (SM).^19^ Overdiagnosis of thyroid nodules results in unnecessary thyroid surgery with associated surgical costs and morbidities.^22–24^ On the other hand, underdiagnosis of thyroid nodules results in delayed treatment, possible reoperation, or more aggressive treatment because of disease progression.^15,17,25^ Therefore, indeterminate thyroid nodules challenge clinicians to plot a course for the optimal management of thyroid nodules.

Although various oncogenes and immunochemical markers have been proposed and studied as diagnostic/prognostic markers for thyroid cancer, their clinical significance has not been generally accepted.^26–28^ The BRAF V600E mutation (hereafter referred to as “the BRAF mutation”) is the most potent activator of the MAPK pathway.^29–31^ This recently discovered somatic mutation is reported in numerous human cancers with various frequencies^32^ and occurs frequently in thyroid cancer.^33–35^ Among thyroid cancers, the BRAF mutation has been identified only in PTC and some apparently PTC-derived anaplastic thyroid carcinoma (ATC), but not in follicular thyroid carcinoma (FTC), medullary thyroid carcinoma (MTC), or BN neoplasms.^35^ Moreover, in patients with PTC, the BRAF mutation is associated with poor clinicopathological outcomes such as advanced tumor stage, extrathyroidal extension, lymph node metastasis, and recurrence.^31,36^ The association between PTC and the BRAF mutation is consistently seen in studies with patients from different geographical and ethnic backgrounds.^31^ These results strongly support a unique function for the BRAF mutation in PTC pathogenesis. On the basis of the high specificity of the BRAF mutation for PTC, several studies have proven the usefulness of the BRAF mutation for identifying PTC from indeterminate cytological results.^28,37–43^ However, in the general population, testing for the BRAF mutation alone might not be sufficient for evaluating thyroid nodules because of its relatively low sensitivity, approximately 44%, for the diagnosis of PTC.^35^

To overcome and supplement the individual shortcomings of US-guided FNAB and BRAF mutation analysis, some previous investigators have tried to predict the probability of PTC by combining them.^28,38–50^ However, these studies were not personalized or quantified for the prediction of PTC probability. Therefore, novel strategies are clearly needed to distinguish PTC from other thyroid nodules and provide personalized and quantitative information about the probability of PTC in the preoperative period. The goal of this study was to design a prediction table and nomogram as tools to preoperatively estimate the probability of PTC using a combination of US, FNAB, and BRAF mutation analysis. We sought to develop 2 distinct prediction models that would enable clinicians and patients to easily personalize and quantify the probability of PTC. Furthermore, we compared 3 molecular methods of BRAF mutation analysis to assess their clinical usefulness and diagnostic accuracy.

## METHODS

### Patient Selection

This study was approved by the Institutional Review Board at Samsung Medical Center, Seoul, South Korea. We retrospectively reviewed the records of 3297 patients who underwent thyroidectomy for thyroid nodules, including malignancies, at the Thyroid Cancer Center of Samsung Medical Center from January 2008 to December 2012. The highest-risk thyroid nodule in US was selected from each patient for analysis. We divided the patients into 2 subgroups, PTC and non-PTC (BN conditions and other types of thyroid cancer except PTC), based on histopathological examinations and then analyzed clinicopathological characteristics of the patients. Within the reviewing period, particularly for patients with a history of completion thyroidectomy for subsequently detected thyroid nodules after initial surgery, we chose the primarily detected nodule.

### Ultrasonography

The primary assessment of thyroid nodules was performed by US scanners (HDI 5000 or IU22; Philips Medical Systems, Bothell, WA) equipped with a commercially available 7 to 12-MHz linear-array transducer. US images were interpreted by ≥2 experienced radiologists for nodule size, shape, echogenicity, margin characteristics, the presence of calcifications, and vascularity. US-detectable thyroid nodules were categorized as low risk (LR), intermediate risk (IR), or high risk (HR).^51–53^ The criteria for HR on US included at least 1 of the following: a taller than wide shape, marked hypoechogenicity, spiculated margins, and microcalcifications or macrocalcifications. In contrast, simple cysts, predominantly cystic nodules with reverberating artifacts, and nodules with a spongiform appearance (especially with intervening isoechoic parenchyma) were defined as LR nodules. IR nodules include nodules having US findings with neither MN nor BN features. The US features for an IR thyroid nodule include isoechogenicity or hyperechogenicity, ovoid-to-round or irregular shape, smooth or ill-defined margin, and rim calcification. When a patient had multiple results of US because of follow-up studies, we selected the result closest to the operation date.

### Fine-Needle Aspiration Biopsy With Cytological Analysis

All patients underwent US-guided FNAB by an experienced radiologist who specialized in thyroid US and its interpretation. Another experienced radiologist reviewed and confirmed the procedures. FNABs were performed when the nodule was >0.5 cm and showed indeterminate or suspicious MN findings on US and smaller nodule accompanied lymph node enlargement. We also considered FNABs in growing BN nodules. For patients with multiple thyroid nodules, the highest-risk thyroid nodule was selected for FNAB. To select the highest-risk thyroid nodule, we primarily consider the nodule character on US. And if some nodules showed similar character on US, then the largest one was targeted for FNAB. US-guided FNAB was performed with a 22 or 23-gauge needle attached to a 2-mL disposable plastic syringe. Aspirates were spread onto glass slides and immediately fixed in 95% alcohol for both Papanicolaou staining and May-Grunwald-Giemsa staining. The criterion for an adequate smear was the presence of 6 groups of cells with >10 cells per group. Based on the TBST,^19^ ≥2 experienced pathologists reviewed slides and classified them as follows: BN, ND/UNS, AUS/FLUS, FN/SFN, SM, or MN. When a patient's record had multiple results of FNAB because of follow-up studies, we selected the result closest to the operation date.

### BRAF Mutation Analysis

BRAF mutation analysis was performed at the Molecular Diagnostics Laboratory of Samsung Medical Center. DNA samples for molecular analysis were extracted from preoperative FNAB specimens or postoperative surgical specimens using QIAamp DNA minikits (QIAGEN, Chatsworth, CA). Three distinct molecular methods were used for molecular analysis of the BRAF mutation. Direct sequencing (DS) after conventional polymerase chain reaction (PCR) was performed in an ABI PRISM 3100 sequencer using BigDye Terminator Cycle Sequencing Ready Reaction Kits (Applied Biosystems, Foster City, CA). Dual priming oligonucleotide (DPO)-based allele-specific PCR (AS-PCR) used the Seeplex BRAF ACE detection system (Seegene, Seoul, Korea) with amplified products analyzed using the ScreenTape system (Lab901 Ltd., Edinburgh, Scotland, UK). Mutant enrichment with 3’-modified oligonucleotides (MEMOs)-based real-time PCR (RT-PCR) used Real-Q BRAF V600E detection kits (BioSewoom, Seoul, Korea) with amplified products analyzed by BigDye Terminator Cycle Sequencing Kits v.3.1 (Applied Biosystems). DNA sequences from all 3 methods were compared with the normal *BRAF* gene exon 15 in the GenBank Database using sequence assembly software (Gene Codes Corp, Ann Arbor, MI). If a thyroid nodule evaluated by ≥2 molecular methods showed different results, we chose and analyzed the positive result of the BRAF mutation.

### Surgical Specimens With Histopathological Examination

Surgical specimens were microscopically examined by ≥2 experienced pathologists and assessed for the following factors: cell type of main lesion, tumor size (measured the longest diameter of the largest lesion), location, multifocality, extrathyroidal extension, lymphovascular invasion, margin involvement, lymph node metastasis, and underlying thyroid condition such as chronic lymphocytic thyroiditis (CLT). Nodule-by-nodule analysis was performed by comparing US and histopathological findings of thyroid nodules including nodule size, location, and characteristics.

### Statistical Analysis

Statistical analysis was performed with SPSS version 21.0 software (IBM, Chicago, IL), and a *P* value <0.05 was defined as statistically significant. Continuous variables were presented as mean ± standard deviation and categorical variables as the number of cases, percent (%), and odds ratio (OR). Chi-square and Fisher exact tests were used for categorical variables and Student *t* test for continuous variables. For the nomogram, multiple logistic regression analysis was used to calculate regression coefficients and internal validation was performed by 10-fold cross-validation. The receiver-operating characteristic (ROC) curve was analyzed to identify the optimal cutoff value of the nomogram points for the diagnosis of PTC. Diagnostic values were sensitivity, specificity, positive predictive value (PPV), negative predictive value, and accuracy. To calculate the diagnostic values of the molecular methods for BRAF mutation analysis, we used the following definitions: true positive—BRAF-positive and PTC by histopathology; true negative—BRAF-negative and non-PTC (BN and other thyroid cancer) by histopathology; false positive—BRAF-positive and non-PTC by histopathology; and false negative—BRAF-negative and PTC by histopathology.

## RESULTS

### Clinicopathological Characteristics of PTC

Histopathological examination of surgical specimens from 3297 thyroid nodules yielded 3107 PTCs and 190 non-PTCs including 36 FTCs, 18 MTCs, 3 ATCs, and 133 BN conditions (Table 1). PTC cases had significantly more total thyroidectomy (OR = 6.205, *P* < 0.001), central neck dissection (OR = 27.435, *P* < 0.001), modified radical neck dissection (OR = 1.883, *P* = 0.034), and open surgery (OR = 1.733, *P* = 0.001). PTC was significantly associated with MN features of FNAB and US (*P* < 0.001), positive BRAF mutation (OR = 39.463, *P* < 0.001), and the presence of CLT (OR = 1.609, *P* = 0.012). Of the 2041 thyroid nodules with MN FNAB, 2034 (99.2%) of them were eventually diagnosed as PTC, whereas the remaining 17 nodules were eventually diagnosed as non-PTC, including 16 MTCs and 1 FTC. Of the 875 thyroid nodules with SM FNAB, 866 (99.0%) of them were eventually diagnosed as PTC, and the remaining 9 nodules were eventually diagnosed as non-PTC, including 3 FTCs, 1 MTC, 1 ATC, and 4 BN conditions. Of the 2382 thyroid nodules with HR US, 2352 (98.7%) of them were eventually diagnosed as PTC, whereas the remaining 30 nodules were eventually diagnosed as non-PTC, including 10 MTCs, 5 FTCs, 3 ATCs, and 12 BN conditions. The BRAF mutation was detected in 2530 (81.4%) of the 3107 PTCs. Of the 2549 thyroid nodules positive for the BRAF mutation, 2530 (99.3%) of them were PTC and the remaining 19 nodules were eventually diagnosed as 2 FTCs, 2 MTCs, and 15 BN conditions.

**TABLE 1:**
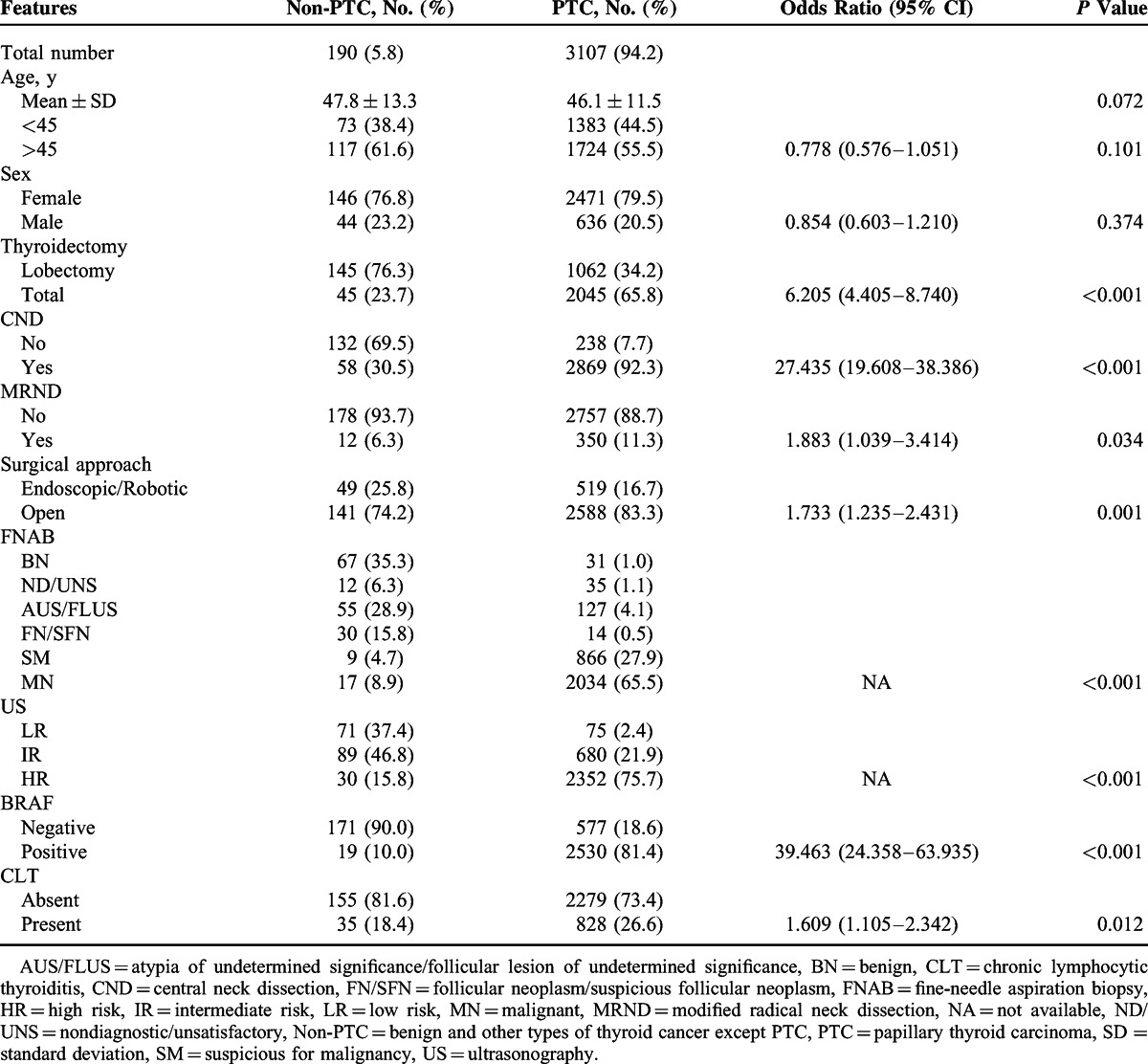
Clinicopathological Characteristics of PTC

### Prediction Table Combining US, FNAB, and BRAF Mutation to Estimate the Probability of PTC

Based on our prediction table (Table 2), the probabilities of PTC for BRAF-positive thyroid nodules were higher than BRAF-negative thyroid nodules, except for thyroid nodules with ND/UNS FNAB and IR US. Although the overall probabilities of PTC for thyroid nodules with MN US and FNAB findings were almost 100% (HR US, 98.7%; SM FNAB, 99.0%; MN FNAB, 99.2%, respectively), the probabilities of PTC according to each diagnostic parameter for US and FNAB were different according to BRAF mutation status. In particular, thyroid nodules with IR US and AUS/FLUS FNAB exhibited a significantly different probability of PTC according to BRAF mutation status (negative, 29.2% vs positive, 87.5%; *P* < 0.001).

**TABLE 2:**
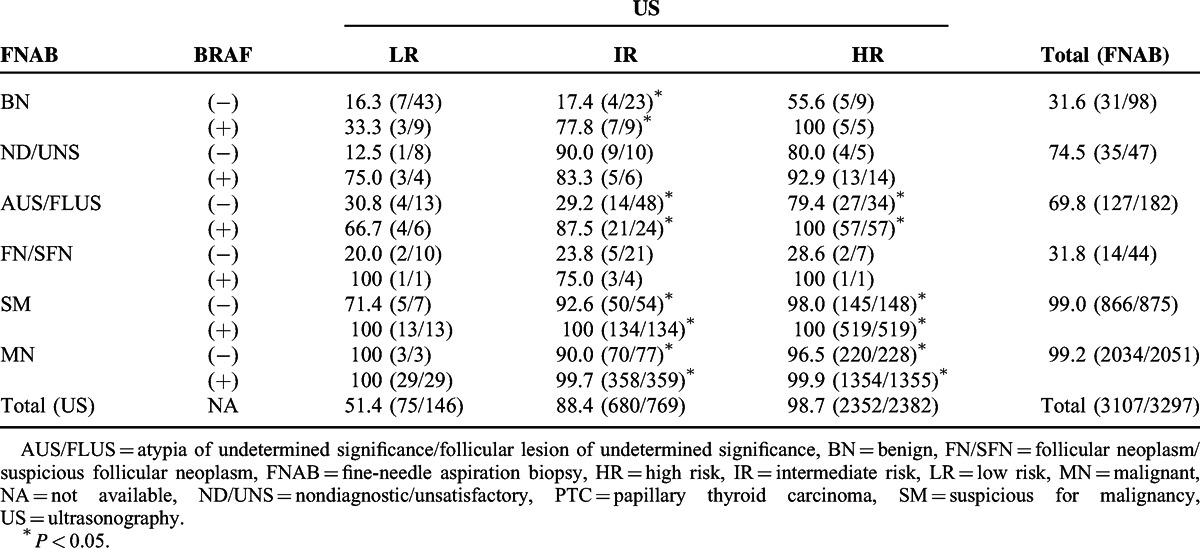
Prediction Table to Estimate the Probability of PTC (%) According to US, FNAB, and BRAF Mutation Status

### Nomogram Combining US, FNAB, and BRAF Mutation to Estimate the Probability of PTC

**FIGURE 1:**
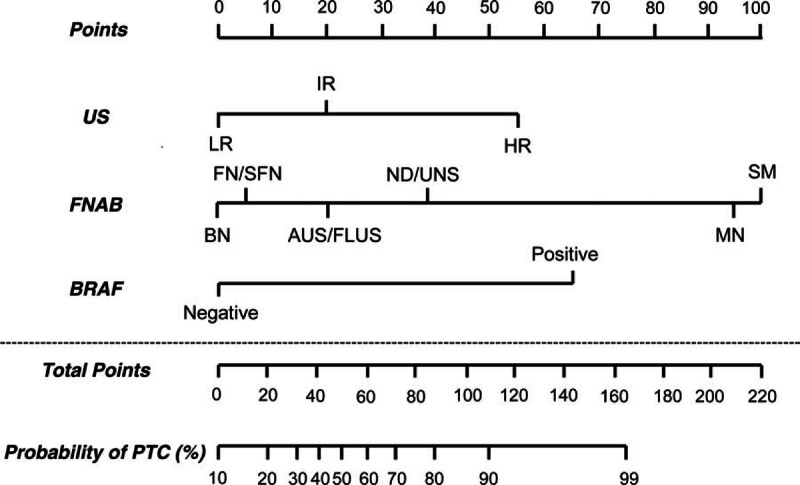
Nomogram to estimate the probability of PTC according to US, FNAB, and BRAF mutation status. AUS/FLUS = atypia of undetermined significance/follicular lesion of undetermined significance, BN = benign, FN/SFN = follicular neoplasm/suspicious follicular neoplasm, FNAB = fine-needle aspiration biopsy, HR = high risk, IR = intermediate risk, LR = low risk, MN = malignant, ND/UNS = nondiagnostic/unsatisfactory, PTC = papillary thyroid carcinoma, SM = suspicious for malignancy, US = ultrasonography.

To design a nomogram, multiple logistic regression analysis was used to calculate regression coefficients of the diagnostic parameters for US, FNAB, and the BRAF mutation (Table 3). The diagnostic parameters of US, FNAB, and the BRAF mutation were proportionally assigned as points on a scale from 0 to 100 in the nomogram by their regression coefficient for PTC (Figure 1). The assigned points of the parameters were US (LR, 0; IR, 20; HR, 56), FNAB (BN, 0; ND/UNS, 35; AUS/FLUS, 20; FN/SFN, 3; SM, 100; MN, 94), and the BRAF mutation (negative, 0; positive, 64). The predicted probability of PTC could be estimated by linearly comparing the total points calculated by the sum of points for the parameters. For example, total nomogram points for thyroid nodules with IR US, AUS/FLUS FNAB, and positive BRAF mutation were 105 [20 (IR US) + 20 (AUS/FLUS FNAB) + 65 (positive BRAF)] and the predicted probability of PTC was approximately 85% to 90%.

**TABLE 3:**
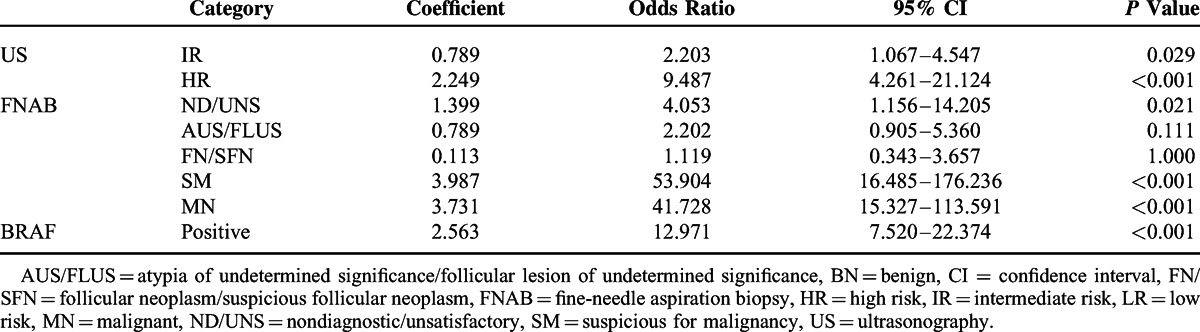
Multiple Logistic Regression Analysis for Diagnostic Parameters of US, FNAB, and BRAF Mutation

### Diagnostic Values of BRAF Mutation Analysis

The BRAF mutation is well known for a high specificity and PPV for PTC.^8^ However, in our study, the overall specificity of BRAF mutation analysis was 90.1% and PPV was 99.3% (Table 4). BRAF mutation analysis was performed by 3 distinct molecular methods: DS after conventional PCR, DPO-based AS-PCR, and MEMO-based RT-PCR. As only 181 patients were evaluated by all the 3 methods, we lacked sufficient numbers for statistical analysis and so only calculated simple diagnostic values. The sensitivity (86.1%), PPV (99.9%), and accuracy (86.3%) of RT-PCR were superior to the other methods. The sensitivity of AS-PCR was higher than that of direct sequencing (77.1% vs 73.2%, respectively), but the specificity of AS-PCR was lower (91.5% vs 95.0%, respectively).

**TABLE 4:**
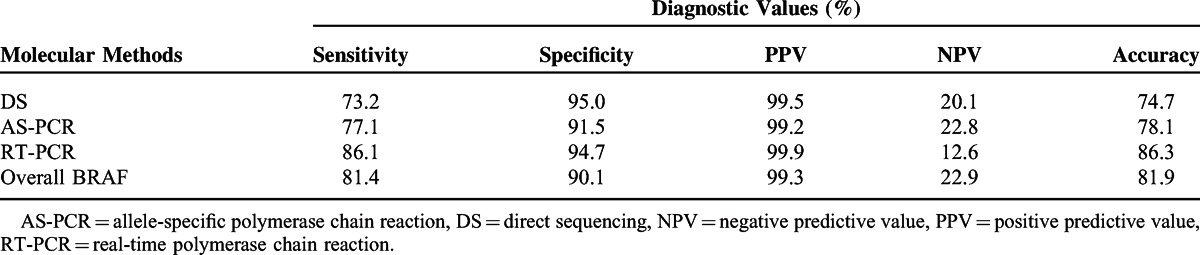
Diagnostic Values of Molecular Methods for BRAF Mutation Analysis

## DISCUSSION

This study designed 2 distinct prediction models for preoperatively predicting the probability of PTC and compared the diagnostic values of 3 molecular methods for BRAF mutation analysis. As reported in the previous studies, 10% to 40% of all US-guided FNAB samples yield indeterminate cytological findings^20,21^ and BRAF mutation analysis shows relatively low sensitivity for the diagnosis of PTC.^35^ Our prediction models clarify the probability of PTC in thyroid nodules with indeterminate FNAB and complement the low sensitivity of BRAF mutation analysis. Either the prediction table (Table 2) or the nomogram (Figure 1) developed in this study can be used by clinicians and patients to estimate the probability of PTC in the preoperative period.

The BRAF mutation was detected in 2530 (81.4%) of 3107 cases of PTC (Table 1). This high prevalence of the BRAF mutation in PTC reflects a geographic bias toward BRAF-prevalent areas.^54–56^ Numerous investigators have reported high specificity and PPV of the BRAF mutation for detecting PTC, ^35^ and we confirmed both high specificity (171/190, 90.1%) and PPV (2530/2549, 99.3%) of the BRAF mutation for PTC. Moreover, the MN features of US and FNAB were significantly more frequent in PTC than in non-PTC. On the basis of these strong relationships of PTC with US, FNAB, and the BRAF mutation, we hypothesize that these parameters could act as predictors for the probability of PTC. In particular, the presence of CLT was significantly associated with PTC, supporting the pathogenesis of PTC associated with preexisting CLT.^57–59^

This study proved the clinical usefulness of BRAF mutation analysis for improved diagnosis of indeterminate thyroid nodules, as suggested by several previous studies.^28,37–42^ As seen in Table 2, the probability of PTC for thyroid nodules with IR US and AUS/FLUS FNAB was largely dependent on BRAF mutation status. Based on this finding, clinicians should perform routine BRAF mutation analysis in indeterminate thyroid nodules because of the HR of PTC in BRAF-positive indeterminate thyroid nodules. In contrast, thyroid nodules with MN findings in US and FNAB showed almost 100% probabilities for PTC regardless of BRAF mutation status. Among the 3026 SM and MN FNAB nodules, only 26 nodules were false positives (including 17 MTCs, 4 FTCs, 1 ATC, and 4 BN conditions). Among the 2382 HR US nodules, only 30 nodules were false positives (including 10 MTCs, 5 FTCs, 3 ATCs, and 12 BN conditions). As 22 (17 MTCs, 4 FTCs, and 1 ATC) of the 26 false positives in FNAB and 18 (10 MTCs, 5 FTCs, and 3 ATCs) of the 30 false positives in US were eventually proven to be MN, clinicians should consider surgical management in thyroid nodules with MN findings in US and FNAB regardless of BRAF mutation status. In thyroid nodules with ND/UNS FNAB and IR US, the probability of PTC for BRAF-negative nodules was higher than for BRAF-positive nodules. This finding reflects heterogeneous pathologic characteristics and indefinite clinical significance of ND/UNS FNAB.^19^

From the TBST,^19^ the risk of malignancy by diagnostic categories is as follows: 0% to 3% for BN, 1% to 4% for ND/UNS, 5% to 15% for AUS/FLUS, 15% to 30% for FN/SFN, 60% to 75% for SM, and 97% to 99% for MN. However, the predicted risk for PTC in the FNAB category MN was lower than the FNAB category SM [(coefficient = 3.731, OR = 41.728) vs (coefficient = 3.987, OR = 53.904), respectively] in our multivariate analysis (Table 3). In our univariate analysis, the FNAB category MN showed a higher risk for PTC than the FNAB category SM (OR = 258.592 vs OR = 207.964, respectively), and some previous studies suggest that MN features of US^40,48^ and positive BRAF mutation^50,60^ are significantly more associated with the FNAB category MN than SM. Thus, the discrepancy in risk for PTC between the FNAB categories SM and MN may be explained by the confounding effects of US and the BRAF mutation in the multivariate analysis. On the other hand, the predicted risk for PTC in the FNAB category FN/SFN was lower than the FNAB category AUS/FLUS [(coefficient = 0.113, OR = 0.119) vs (coefficient = 0.789, OR = 2.202), respectively] in our multivariate analysis. The discrepancy in risk for PTC between the FNAB category FN/SFN and AUS/FLUS may be explained by a difference in the definition of “Gold Standard” between the TBST and this study. In the TBST, the purpose of FN/SFN is to identify suspicious nodules for FTC. In our study, however, FTC, MTC, ATC, and other thyroid cancers except PTC were categorized as non-PTC, the true negative.

As seen in Figure 1, we designed a unique and interesting nomogram for the diagnosis of PTC. The predictive power of the nomogram was verified by ROC curve and internal validation. Area under the ROC curve was >0.9 for both the original sample [0.970 (0.957–0.983)] and internal validation [0.969 (0.955–0.981)], suggesting that the nomogram has a high discrimination ability for PTC.

We also compared 3 distinct molecular methods for diagnostic accuracy of BRAF mutation analysis (Table 4). In agreement with several recent studies,^61,62^ we demonstrated that RT-PCR is a more reliable method than the other 2 molecular methods. In particular, as shown in Table 1, there were 19 BRAF-positive non-PTC thyroid nodules, including 2 FTCs, 2 MTCs, and 15 BN conditions. Of these 19 false-positive nodules, only 1 thyroid nodule was analyzed by RT-PCR and the remaining 18 nodules were analyzed by DS and/or AS-PCR. Accordingly, the false positives of the BRAF mutation in this study may be due to the low diagnostic accuracy of DS and AS-PCR. Although our DNA samples for BRAF mutation analysis were extracted from both FNAB specimens and surgical specimens, numerous studies have demonstrated that BRAF mutation analysis can be performed readily and reliably in FNAB specimens, preoperatively.^52,53^

This study had several limitations. First, because this was a retrospective study, patient information might not have been fully collected at the time of treatment. Second, this study was conducted in a BRAF-prevalent area and so these results might not be applicable to other countries or races. Third, the selection of cases was biased to patients who underwent thyroidectomy. We did not include patients with truly BN conditions who were followed in an outpatient clinic without surgery. Fourth, we did not perform BRAF mutation analysis as a routine preoperative examination, and molecular methods have changed over the years. Consequently, 3 distinct molecular methods and 2 distinct specimen types were used during the period in review, which may have led to discrepancies in the results of the molecular analysis. Fifth, due to a lack of data, statistical analysis could not be performed when comparing molecular methods for BRAF mutation analysis. Sixth, since the BRAF mutation is only found in PTCs, the prediction models cannot be applied to other types of thyroid cancers. Seventh, one of the major limitations of this study is the small number of indeterminate results upon FNAB. Thus, the use of the prediction model to identify PTC from relatively small portion of indeterminate nodules requires large-scaled prospective study and should be considered selectively. Finally, interobserver variability might have occurred for the interpretation of US and FNAB. At the same time, the use of surgically proven data and a large sample size are strengths of our study.

In conclusion, we strongly recommend the use of the prediction table and nomogram developed in this study. This prediction model enables patients and clinicians to easily assess the probability of PTC in preoperative period and provides personalized and quantified information of the probability of PTC in variable situations. Furthermore, preoperative BRAF mutation analysis combined with US and FNAB findings could increase the diagnostic accuracy of PTC in indeterminate thyroid nodules. However, BRAF mutation analysis may give little additive value for the diagnosis of thyroid nodules that have suspicious MN or MN findings on preoperative US or FNAB. Further prospective investigation will be required to support our prediction models. Somatic mutation testing, mRNA gene expression platforms, protein immunocytochemistry, and microRNA panels have improved the diagnostic accuracy of indeterminate thyroid nodules, although no test is perfectly accurate.^63^ As molecular biology advances, we expect that molecular tests will be developed that will further enable us to readily classify thyroid nodules in the preoperative period.
